# Safety and effectiveness of Omnitrope^®^ (somatropin) in PATRO Children: a multi-center, post-marketing surveillance study comparison of US and international cohort data

**DOI:** 10.1007/s00431-022-04409-8

**Published:** 2022-03-11

**Authors:** Philippe Backeljauw, Shankar Kanumakala, Sandro Loche, Karl Otfried Schwab, Bradley S. Miller, Richard Levy, Kenneth McCormick, Hichem Zouater, Markus Zabransky, Kim Campbell

**Affiliations:** 1 Cincinnati Children’s Hospital Medical Center, University of Cincinnati College of Medicine, Cincinnati, OH USA; 2 Royal Alexandra Children’s Hospital, University Hospitals Sussex NHS Trust, Brighton, UK; 3 Ospedale Pediatrico Microcitemico “A. Cao”, AO Brotzu, Cagliari, Italy; 4 Department of Pediatrics and Adolescent Medicine, Faculty of Medicine, University of Freiburg, Freiburg, Germany; 5 University of Minnesota Masonic Children’s Hospital, Minneapolis, MN USA; 6 Rush University Medical Center, Chicago, IL USA; 7 Division of Endocrinology, University of Alabama at Birmingham, Birmingham, AL USA; 8 Sandoz Biopharmaceuticals, c/o HEXAL AG, Holzkirchen, Germany; 9 Sandoz Inc, Princeton, NJ USA

**Keywords:** Pediatrics, Growth hormone, Omnitrope®, PATRO Children, Non-interventional study

## Abstract

There are known geographical differences in growth hormone deficiency (GHD) patient populations and treatment practices. Here, we present a comparison of safety and effectiveness data from patients treated with recombinant human growth hormone (rhGH) in the USA versus other countries. PAtients TReated with Omnitrope® (PATRO) Children is an international, non-interventional study with Omnitrope® (somatropin, Sandoz Inc.). All visits and assessments are carried out according to routine clinical practice, and doses of Omnitrope® are given according to country-specific prescribing information. By September 2018, 294 patients had been enrolled in the USA (53% rhGH-naïve) and 6206 patients had been enrolled across 13 other countries (international group; 86% rhGH-naïve). The most common indication in both groups was GHD. Overall, 194 US patients (66%) and 2977 international patients (48%) experienced adverse events (AEs; 886 and 11,716 events, respectively), most of which were of mild or moderate intensity. The AEs were suspected to be treatment-related in five US patients (1.7%) and 452 international patients (7.3%). All reported neoplasms were benign, non-serious, and considered unrelated to rhGH therapy. No cases of diabetes mellitus or hyperglycemia were reported. In rhGH-naïve GHD patients, after 3 years of rhGH therapy, the improvement in mean height SD score from baseline was + 1.25 and + 1.35 in US and international patients, respectively.

*Conclusion*: Omnitrope® treatment appears to be well tolerated and effective in US patients and those from other countries. Across the pediatric indications included, there was no evidence of an increased risk of developing uncommon or unexpected AEs with rhGH.

*Trial registration*: NA.

**Table:** 

**What is Known:** *• Continued monitoring of patients treated with recombinant human growth hormone (rhGH) is important, particularly in terms of diabetogenic potential and the risk of malignancies.* *• The PAtients TReated with Omnitrope® (PATRO) Children study is a long-term, post-marketing surveillance program for the rhGH Omnitrope®*.	
**What is New:** *• Omnitrope® is well tolerated and effective in US patients, and those from other countries.* *• Across all indications included, there were no unexpected adverse events and there was no evidence of an increased risk of developing malignancies or diabetes.*

**What is Known:**

*• Continued monitoring of patients treated with recombinant human growth hormone (rhGH) is important, particularly in terms of diabetogenic potential and the risk of malignancies.*

*• The PAtients TReated with Omnitrope® (PATRO) Children study is a long-term, post-marketing surveillance program for the rhGH Omnitrope®*.

**What is New:**

*• Omnitrope® is well tolerated and effective in US patients, and those from other countries.*

*• Across all indications included, there were no unexpected adverse events and there was no evidence of an increased risk of developing malignancies or diabetes.*

## Introduction

Omnitrope® is a recombinant human growth hormone (rhGH; somatropin) approved in the USA and in Europe in 2006 [1–3]. In Europe, Omnitrope® was approved as biosimilar on the basis that it matches the reference medicine (Genotropin®, Pfizer) in terms of safety, efficacy, and quality [4]. Approval of Omnitrope® in other countries has since followed [1, 5]. The PAtients TReated with Omnitrope® (PATRO) Children study is a long-term, post-marketing surveillance program for Omnitrope®, initiated in 2006 [6, 7].

Approved pediatric indications for rhGH include children with growth hormone deficiency (GHD), Turner syndrome, Prader-Willi syndrome (PWS), and short children born small for gestational age (SGA) [8, 9]. A position statement supported by several endocrinology societies and published in 2016 concluded that rhGH has a good safety record when used to treat approved indications at recommended doses [10]. Nevertheless, the statement recognized the importance of continued monitoring of patients treated with rhGH [10], highlighting the value of post-approval surveillance studies such as PATRO Children.

Previous research has demonstrated the involvement of rhGH in glucose level regulation, impaired glucose metabolism, and insulin resistance. Subsequently, the risk of diabetes mellitus in adult GHD patients is increased compared with the general population, particularly in those with additional risk factors, such as a family history of diabetes mellitus or obesity [11, 12]. Studies from real-life clinical practice have explored the impact of rhGH treatment on glucose metabolism in GHD patients. From this, no signals of increased risk for diabetes mellitus were observed; however, continued follow-up is still imperative [11]. Additionally, multiple lines of evidence have suggested that GH and insulin-like growth factor (IGF-I) can influence cancer incidence and progression. Since rhGH therapy increases levels of IGF-I, there has been a concern around its potential to influence the risk of malignancies and neoplastic tissue growth [12–14]. Long-term, international cohort investigations, such as the SAGhE study, have demonstrated results that do not suggest a potential carcinogenic effect of rhGH. However, there is still uncertainty and thus requires further investigation [15]. As a result, these adverse events (AEs) were noted as special interest and continuously monitored for in both patient subgroups.

The main objective of PATRO Children is to assess the safety of rhGH in pediatric patients, particularly in terms of diabetogenic potential and the risk of malignancies in all indications. The observed patient populations, referral patterns, and treatment practices are known to vary in the USA compared with other countries. Therefore, the objective was to utilize the US cohort as a proxy for these variables and assess whether Omnitrope® treatment is similarly well tolerated and effective in patients enrolled from the US and international cohorts, despite these differences. The effectiveness of rhGH is analyzed as a secondary objective [6, 7]. Here, we present a descriptive comparison of safety and effectiveness data from patients enrolled in the USA and patients enrolled in other countries, using data from an analysis conducted in September 2018.

## Materials and methods

PATRO Children is an international, longitudinal, non-interventional, observational study conducted in hospitals and specialized endocrinology clinics across 14 countries. The study methodology has previously been described [6, 7]. In brief, infants, children, and adolescents who require rhGH treatment and receive at least one dose of Omnitrope® are enrolled. Patients previously treated with another rhGH medicine prior to starting the study are also eligible. Omnitrope® is administered per standard clinical practice and doses are given according to country-specific prescribing information chosen by the physician.

### Safety assessments

AEs are monitored and recorded for the duration of Omnitrope® treatment. Emphasis is placed on long-term safety, recurrence or new onset of malignancies, and the development of glucose intolerance or diabetes. The seriousness and relationship of AEs to study treatment are independently evaluated by investigator and sponsor assessment, and classified according to worst-case scenario. The intensity of AEs (mild, moderate, severe) is assessed by the investigator. Laboratory values, including glucose metabolism and anti-human growth hormone antibodies, are requested to be reported at least once a year, if obtained. Reasons for treatment discontinuation are also recorded.

### Effectiveness assessments

Auxological data may be recorded at each visit and are requested to be documented at least once a year. Height velocity (HV, cm/year), height standard deviation (SD) score (HSDS), HV SD score (HVSDS), and body mass index (BMI) SD score (SDS) are derived from height and weight measurements and country-specific reference tables.

### Data collection and statistical analysis

Patient data are recorded in an electronic case report form (eCRF) at each visit. The eCRFs are reviewed and monitoring is performed by a contract research organization. Standard descriptive statistics are used to describe continuous parameters (e.g., age, height, weight) and categorical parameters (e.g., sex); as a non-interventional, observational study, statistical comparisons of safety and efficacy data were not possible.

The safety population includes all patients documented in the eCRF before the cut-off date (September 2018). The effectiveness population is a subset of the safety population and includes all patients with a documented height measurement at baseline (start of Omnitrope® treatment) and ≥ 1 measurement of height during study treatment (≥ 60 days after baseline). The 3-year analysis set includes patients who have completed at least 3 years of Omnitrope® treatment.

In the current analysis, data from patients enrolled in the USA are compared with the combined data from patients enrolled in the following countries: Austria, Canada, Czech Republic, France, Germany, Italy, Poland, Romania, Slovenia, Spain, Sweden, Taiwan, and the UK (the international group).

## Results

### Patients and treatment (safety population)

As of September 2018, 294 patients had been enrolled from 14 centers in the USA (US group) and 6206 patients had been enrolled from 299 centers across 13 other countries (international group). The most common indication in both was GHD; other indications are shown in Table 1.

**Table 1:** Recruitment per indication (safety population)

**Indication**	**Patients, *n* (%)**	
	**US**	**International**
GHD	193 (66)	3571 (58)
SGA	8 (2.7)	1669 (27)
TS	9 (3.1)	309 (5.0)
PWS	2 (0.7)	221 (3.6)
ISS^a^	62 (21)	135 (2.2)
CRI	0	60 (1.0)
Other	20 (6.8)	225 (3.6)
Unknown	0	16 (0.3)
Total	294 (100)	6206 (100)

*CRI* chronic renal insufficiency, *GHD* growth hormone deficiency, *ISS* idiopathic short stature, *PWS* Prader-Willi syndrome, *SGA* small for gestational age, *TS*, Turner syndrome, *US* United States

^a^Omnitrope® is approved for ISS patients in the USA, Canada, and Brazil only

Mean (SD) age at enrollment (baseline visit) was 10.4 (3.6) years for US patients and 73% of patients were male. For patients in the international group, mean (SD) age at enrollment was 9.0 (3.9) years and 59% of patients were male. Overall, 53% of US patients and 86% of international patients were rhGH-naïve at study entry. In rhGH-naïve patients, the mean (SD) age at study enrollment was 10.8 (3.5) years in US patients and 8.6 (3.9) years in international patients.

The mean duration of Omnitrope® treatment was just over 3 years; mean (SD) 40.2 (17.4) months for US patients and 38.2 (26.9) months for international patients. In total, 201 US patients (68%) and 3433 international patients (55%) completed at least 3 years of Omnitrope® treatment.

For US patients, the mean (SD) prescribed Omnitrope® dose at baseline was 47.3 (14.4) µg/kg/day and by Year 3 in the study, the mean (SD) dose was 54.1 (28.9) µg/kg/day. In rhGH-naïve US patients, the mean (SD) dose was 45.5 (8.3) µg/kg/day at baseline and 56.0 (35.3) µg/kg/day at Year 3. For international patients, the mean (SD) prescribed dose at baseline was 32.1 (9.4) µg/kg/day and by Year 3, the mean (SD) dose was 35.9 (10.6) µg/kg/day. In rhGH-naïve international patients, the mean (SD) dose was 31.7 (9.2) µg/kg/day at baseline and 35.9 (10.4) µg/kg/day at Year 3.

### Safety (safety population)

As of September 2018, 230 US patients (78%) and 2639 international patients (43%) had discontinued the study. The primary reasons for discontinuation are shown in Table 2. AEs were the primary reason for discontinuation for three US patients and 102 international patients (1.3% and 3.9% of discontinued patients, respectively).

**Table 2:** Primary reasons for study discontinuation

**Reason**	**Patients, *n *(%)**		**SMD** **(%)**
	**US**	**International**	
Patient reached adult height/bone age maturation	23 (10)	638 (24)	−38.3
Reached near adult height	9 (3.9)	323 (12)	−30.9
Patient satisfied with current height	18 (7.8)	115 (4.4)	14.5
Miscellaneous reasons	66 (29)^a^	466 (18)^b^	26.4
Lost to follow-up	25 (11)	373 (14)	−9.9
Patient does not wish to continue the injections	17 (7.4)	264 (10)	−9.3
Switch to other rhGH medicine	63 (27)	100 (3.8)	68.8
Non-responder	3 (1.3)	110 (4.2)	−17.6
Adverse event	3 (1.3)	102 (3.9)	−16.2
Patient non-compliant	1 (0.4)	78 (3.0)	−19.6
Referral to adult endocrinologist	1 (0.4)	29 (1.1)	−7.6
HV slowdown (HV < 1 cm/year)	0	16 (0.6)	−11.0
Withdrawal of informed consent^c^	0	13 (0.5)	−10.0
Unknown	1 (0.4)	8 (0.3)	2.2
Indication for Omnitrope® no longer applicable	0	4 (0.2)	−5.5
Total	230 (100)	2639 (100)	

*HV* height velocity, *rhGH* recombinant human growth hormone *SMD* standardized mean difference

^a^Recorded as other reasons, site closure, or insurance reasons

^b^Recorded as other reasons or site closure

^c^Withdrawal of informed consent denotes that the patient withdrew from the study of their own decision (or was withdrawn by their caregiver)

A summary of AEs is provided in Table 3. Overall, 194 US patients (66%, 95% confidence interval [*CI*] 60.3, 71.4) and 2977 international patients (48%, 95% *CI* 46.7, 49.2) experienced AEs (886 and 11,716 events, respectively), most of which were of mild or moderate intensity. AEs leading to discontinuation were considered treatment-related in one US patient (0.3%) and in 57 international patients (0.9%). AEs were suspected to be treatment-related in five US patients (1.7%, 95% *CI* 0.6, 3.9) and 452 international patients (7.3%, 95% *CI* 6.6, 8.0) (Table 3).

**Table 3:** Summary of AEs

	**US** **(*N* = 294)**		**International** **(*N* = 6206)**		**SMD** **(%)**
	**Patients, *n* (%)**	**95% *CI***	**Patients, *n* (%)**	**95% *CI***	
**Any AE**	194 (66)	60.3, 71.4	2977 (48)	46.7, 49.2	37.0
**Relationship to study drug**					
Not suspected	192 (65)	59.6, 70.7	2875 (46)	45.1, 47.6	38.9
Suspected	5 (1.7)	0.6, 3.9	452 (7.3)	6.6, 8.0	−27.2
Missing/not assessable	0	–	15 (0.2)	–	–
**Intensity**					
Mild	145 (49)	–	2307 (37)	–	24.7
Moderate	55 (19)	–	1325 (21)	–	−6.6
Severe	13 (4.4)	–	287 (4.6)	–	−1.0
Missing	130 (44)	–	491 (7.9)	–	–
**Changes to rhGH treatment**					
Not changed	145 (49)	43.5, 55.2	2855 (46)	44.8, 47.3	6.6
Increased	14 (4.8)	2.6, 7.9	110 (1.8)	1.5, 2.1	16.9
Reduced	6 (2.0)	–	72 (1.2)	–	7.0
Interrupted	11 (3.7)	–	172 (2.8)	–	5.5
Permanently discontinued	3 (1.0)	–	105 (1.7)	–	−5.8
Missing	128 (44)	–	24 (0.4)	–	–
**Treatment-related AEs (≥ 15 patients**^a^**), by MedDRA preferred term**					
Headache	0	–	107 (1.7)	–	–
Injection-site pain	0	–	54 (0.9)	–	–
Injection-site hematoma	0	–	37 (0.6)	–	–
Arthralgia	1 (0.3)	–	29 (0.5)	–	–
Hypothyroidism	0	–	19 (0.3)	–	–
Insulin-like growth factor increased	0	–	18 (0.3)	–	–
Sleep apnea syndrome	0	–	17 (0.3)	–	–
Heart rate increased	1 (0.3)	–	0	–	–
Kyphosis	1 (0.3)	–	0	–	–
Overdose	1 (0.3)	–	3 (0.0)	–	–
Swelling face	1 (0.3)	–	0	–	–
**SAEs**					
No	191 (65)	59.2, 70.4	2840 (46)	44.5, 47.0	39.4
Yes	14 (4.8)	2.6, 7.9	738 (12)	11.1, 12.7	−26.0
Missing	0	–	10 (0.2)	–	–
**SAE relationship to study drug**					
Not suspected	13 (4.4)	2.4, 7.4	699 (11)	10.5, 12.1	−25.7
Suspected	1 (0.3)	0, 1.9	49 (0.8)	0.6, 1.0	−6.0
Missing	0	–	1 (0.0)	–	–

*AE* adverse event, *CI* confidence interval, *MedDRA* Medical Dictionary for Regulatory Activities, *rhGH* recombinant human growth hormone, *SAE* serious adverse event, *SMD* standardized mean difference, *US* United States

^a^For international patients only. All treatment-related AEs shown for US cohort

When stratified according to treatment duration, a higher proportion of US patients reported at least one AE compared with international patients for the following treatment durations: ≤ 1 year (US 33% of patients with ≥ 1 AE vs. international 16%); 1 to ≤ 2 years (US 67% vs. international 38%); and 2 to ≤ 3 years (US 75% vs. international 49%). In patients with longer treatment durations (between 3 and 5 years), the percentage of patients experiencing AEs was similar in the US and international groups.

There were 14 US patients (4.8%, 95% *CI* 2.6, 7.9) who experienced serious adverse events (SAEs; *n* = 19 events), including one patient (0.3%, 95% *CI* 0, 1.9) who had a treatment-related SAE. The treatment-related SAE was kyphosis (Medical Dictionary for Regulatory Activities [MedDRA] preferred term), which was reported in a male patient with ISS after 1.4 years of treatment (Table 4). In total, 738 patients (12%, 95% *CI* 11.1, 12.7) from the international group experienced SAEs (*n* = 1429 events), including 49 patients (0.8%, 95% *CI* 0.6, 1.0) who had SAEs that were considered treatment-related. Treatment-related SAEs included events in the following MedDRA system order classes: respiratory, thoracic, and mediastinal disorders (*n* = 16 patients), nervous system disorders (*n* = 7), metabolism and nutrition disorders (*n* = 4), and neoplasms benign, malignant, and unspecified (*n* = 4). Further details for treatment-related SAEs of interest are provided in Table 4.

**Table 4:** Details of patients with treatment-related SAEs of interest

**Group**	**MedDRA system organ class**	**SAE** **(preferred term)**	**Indication, sex, age (years) at** **SAE onset**	**Onset of event after start of Omnitrope® therapy**	**Action taken with treatment**	**Outcome**
US	Musculoskeletal and connective tissue disorders	Kyphosis	ISS, male, 16	1.4 years	Permanently discontinued	Ongoing
International	Respiratory, thoracic, and mediastinal disorders	Sleep apnea syndrome	PWS, male, 3	2.4 years	Not changed	Resolved completely
			PWS, female, 1	0.7 years	Not changed	Resolved completely
			PWS, male, 2	1.1 years	Not changed	Resolved completely
			PWS, male, 4	3.1 years	Not changed	Resolved completely
			PWS, female, 1	0.5 years	Reduced	Resolved completely
			PWS, female, 1	0.6 years	Not changed	Resolved completely
			PWS, male, 1	35 days	Interrupted	Resolved completely
				0.7 years	Reduced	Resolved completely
			PWS, female, 2	0.7 years	Not changed	Resolved completely
				1.0 year	Not changed	Resolved completely
				0.8 years	Not changed	Resolved completely
			PWS, female, 3	1.0 year	Not changed	Ongoing
			PWS, male, NR	NR	Interrupted	Ongoing
		Adenoidal hypertrophy	SGA, male, 6	1.9 years	Not changed	Resolved completely
			PWS, male, 2	1.8 years	Not changed	Resolved completely
			PWS, female, 3	2.4 years	Not changed	Resolved completely
			PWS, female, 1	0.6 years	Not changed	Resolved completely
			PWS, female, 2	0.8 years	Not changed	Resolved completely
	Nervous system disorders	Increased intracranial pressure	GHD, female, 6	32 days	Interrupted	Resolved completely
			SGA, male, 4	1.6 years	Permanently discontinued	Resolved completely
	Metabolism and nutrition disorders	Impaired glucose tolerance	SGA, male, 8	2.1 years	Permanently discontinued	Ongoing
			SGA, male, 11	7.8 years	Permanently discontinued	NR
		Type 1 diabetes	SGA, female, 14	0.8 years	Permanently discontinued	Ongoing
	Neoplasms benign, malignant, and unspecified	Neoplasm progression	GHD, male, 19	5.1 years	Interrupted	Resolved completely
			Other, female, 18	2.3 years	Interrupted	Resolved with sequelae
		Craniopharyngioma	GHD, female, 6	3.9 years	Not changed	Resolved completely

*GHD* growth hormone deficiency, *ISS* idiopathic short stature, *MedDRA* Medical Dictionary for Regulatory Activities, *NR* not recorded, *PWS* Prader-Willi syndrome, *SAE* serious adverse event, *SGA* small for gestational age, *US* United States

Among US patients who received a baseline rhGH dose of ≤ 50 µg/kg/day (*n* = 133), 54% reported at least one AE (*n* = 250 events) and 2.3% had a SAE (*n* = 3 events). Treatment-related AEs were reported in 0.8% of patients (*n* = 1 event). In US patients with a baseline dose > 50 µg/kg/day (*n* = 57), 77% reported an AE (*n* = 285 events) and 3.5% had a SAE (*n* = 3 events). Treatment-related AEs were reported in 3.5% of patients (*n* = 2 events). In patients from the international group who received a baseline rhGH dose of ≤ 35 µg/kg/day (*n* = 3515), 45% had at least one AE (*n* = 6146 events) and 12% had a SAE (*n* = 742 events). Treatment-related AEs were reported in 7.1% of patients (*n* = 364 events). In patients with a baseline dose > 35 µg/kg/day (*n* = 1332), 54% had an AE (*n* = 2895 events) and 13% had a SAE (*n* = 319 events). Treatment-related AEs were reported in 8.3% of patients (*n* = 147 events).

### Effectiveness (3-year effectiveness population)

In total, 146 US patients completed at least 3 years of treatment and were included in the 3-year effectiveness population (including 54 rhGH-naïve GHD patients and 16 rhGH-naïve ISS patients). A total of 2753 international patients were included in the 3-year effectiveness population (including 1407 rhGH-naïve GHD patients and 56 rhGH-naïve ISS patients).

#### GHD patients

**Fig. 1:**
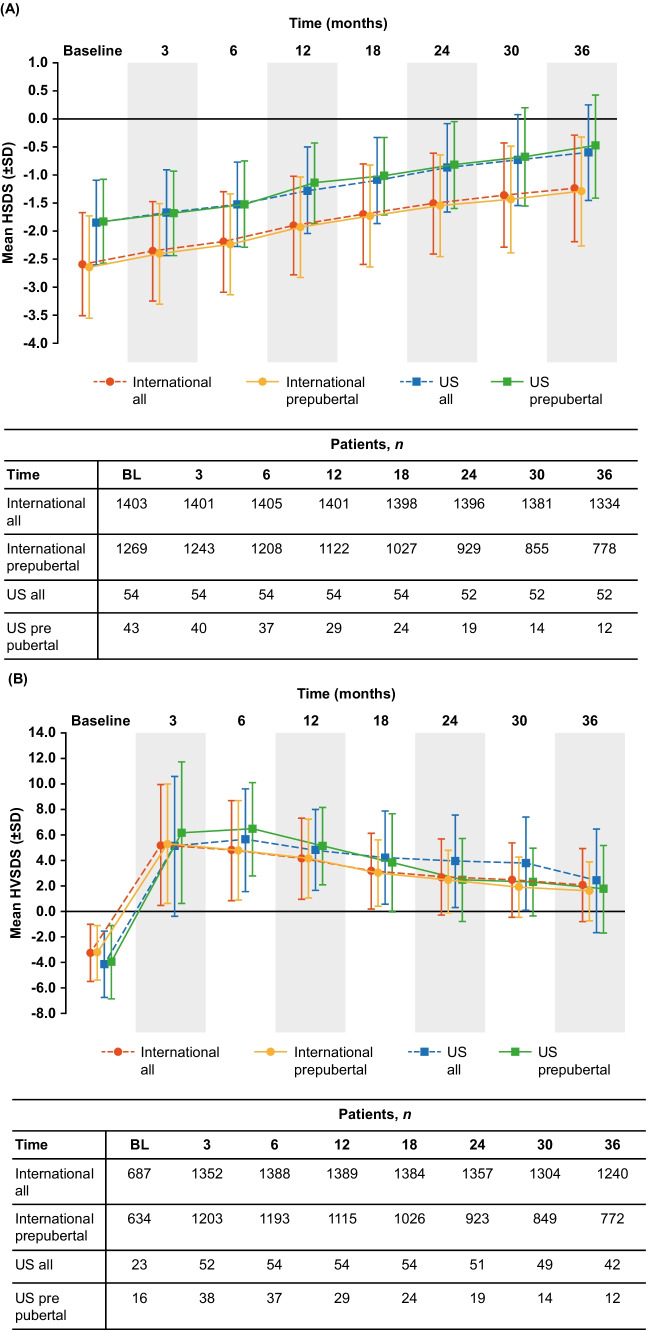
Attained HSDS **(A)** and HVSDS **(B)** for rhGH-naïve GHD patients following 3 years of Omnitrope® treatment. *BL* baseline, *GHD* growth hormone deficiency, *HSDS* height standard deviation score, *HVSDS* height velocity standard deviation score, *rhGH* recombinant human growth hormone, *SD* standard deviation, *SDS* standard deviation score, *US* United States

Improvements in HSDS and peak-centered HVSDS for rhGH-naïve GHD patients over 3 years of Omnitrope® treatment are shown in Fig. 1. In rhGH-naïve GHD patients, the improvement from baseline in mean HSDS at Year 1 was + 0.58 and + 0.69 in US and international patients, respectively. At Year 3, the improvement in mean HSDS from baseline was + 1.25 and + 1.35 in US and international patients, respectively. Similar improvements in HSDS were observed in prepubertal rhGH-naïve GHD patients; at Year 1, the mean improvement was + 0.68 (US patients) and + 0.71 (international patients) and at Year 3, the mean improvement was + 1.34 (US patients) and + 1.35 (international patients) (Fig. 1A).

At Year 3, the improvement from baseline in mean HVSDS was + 6.54 (US patients) and + 5.35 (international patients) in rhGH-naïve GHD patients. In prepubertal rhGH-naïve patients, the improvement in mean HVSDS at Year 3 was + 5.73 in US patients and + 4.82 in international patients (Fig. 1B).

In rhGH-naïve GHD patients from the USA, BMI SDS increased from −0.48 at baseline to −0.13 at Year 3, an improvement of + 0.35. In international GHD patients, BMI SDS increased from −0.22 at baseline to −0.05 at Year 3, an improvement of + 0.17.

In the US group, improvements at 1 year in HSDS (+ 0.63 vs. + 0.59), HVSDS (+ 8.73 vs. + 8.56), and BMI SDS (+ 0.18 vs. + 0.13) were similar in the low baseline-dose group (≤ 50 µg/kg/day) and the high baseline-dose group (> 50 µg/kg/day). For the international group, improvements at 1 year were also similar between the high baseline-dose group (> 35 µg/kg/day) and the low baseline-dose group (≤ 35 µg/kg/day: HSDS, + 0.72 vs. + 0.66; HVSDS, + 7.59 vs. + 7.24; BMI SDS, + 0.05 vs. + 0.01).

#### ISS patients

**Fig. 2:**
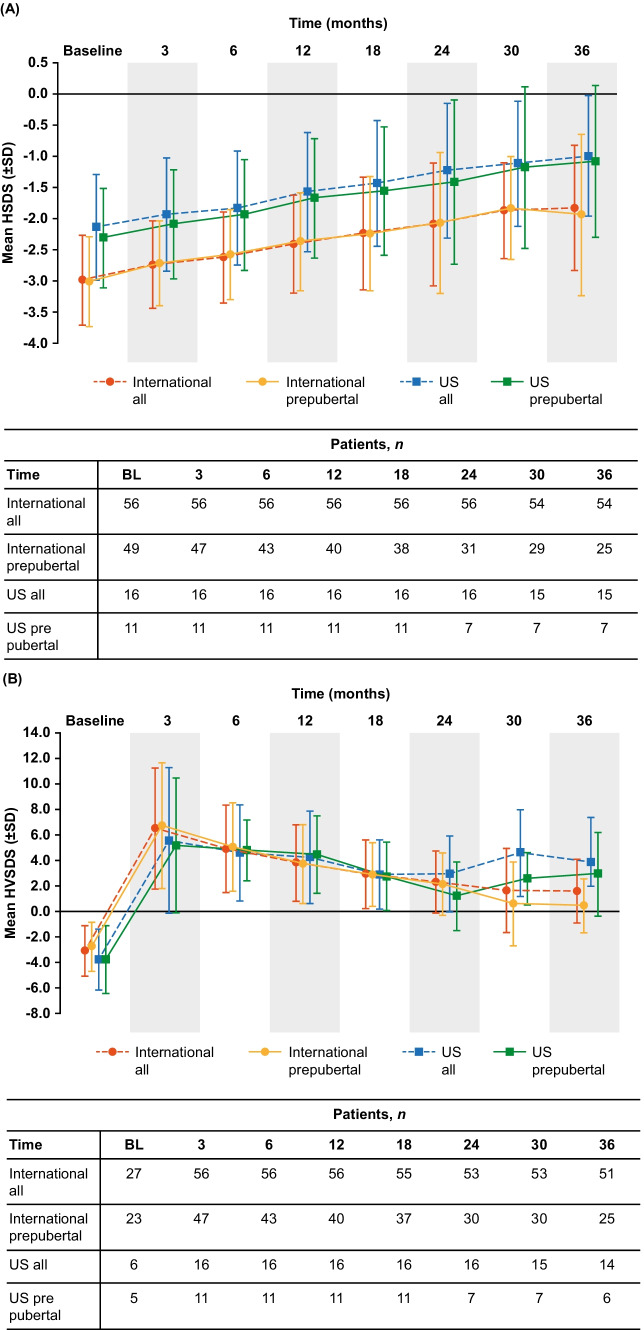
Attained HSDS **(A)** and HVSDS **(B)** for rhGH-naïve ISS patients following 3 years of Omnitrope® treatment. *BL* baseline, *HSDS* height standard deviation score, *HVSDS* height velocity standard deviation score, *ISS* idiopathic short stature, *rhGH* recombinant human growth hormone, *SD* standard deviation, *SDS* standard deviation score, *US* United States

Improvements in HSDS and peak-centered HVSDS for rhGH-naïve ISS patients over 3 years of Omnitrope® treatment are shown in Fig. 2. In ISS patients who were rhGH-naïve at study entry, the improvement from baseline in mean HSDS at Year 1 was + 0.57 (US patients) and + 0.58 (international patients). At Year 3, the improvement in mean HSDS from baseline was + 1.14 (US patients) and + 1.17 (international patients). In prepubertal rhGH-naïve ISS patients, the mean improvement in HSDS at Year 1 was + 0.64 in both US and international groups. At Year 3, the mean improvement in HSDS was + 1.23 (US patients) and + 1.07 (international patients) (Fig. 2A).

In rhGH-naïve ISS patients, the improvement from baseline at Year 3 in mean HVSDS was + 7.63 (US patients) and + 4.67 (international patients). In prepubertal rhGH-naïve ISS patients, the improvement in mean HVSDS at Year 3 was + 6.71 (US patients) and + 3.22 (international patients) (Fig. 2B).

In rhGH-naïve ISS patients from the USA, BMI SDS was 0.33 at baseline and 0.44 at Year 3 (an improvement of + 0.11). In international ISS patients, BMI SDS was −0.58 at baseline and −0.59 at Year 3.

## Discussion

This analysis from the non-interventional, observational PATRO Children study indicates that treatment with Omnitrope® is well tolerated and effective in patients enrolled from the USA and other countries. Some differences were apparent between US and international patients, which likely reflect variations in referral patterns, rhGH treatment practices, and approved indications across the countries in the study.

Overall, the effectiveness findings from PATRO Children are consistent with other large observational studies of rhGH. In an analysis of data from the observational NordiNet® International Outcomes Study and the NovoNet®/American Norditropin® Studies, similar improvements in HSDS were observed following 1 year of rhGH therapy in patients with isolated GHD and ISS [16]. However, in contrast with the data from PATRO Children, slightly higher HSDS gains following 1 year of treatment were observed in prepubertal GHD and ISS patients compared with the overall group for each indication [16]. As participants in the PATRO Children study were, on average, older than those in the NordiNet®/NovoNet® studies, this difference may be due to an effect of age and/or puberty in PATRO Children.

Across the pediatric indications examined in the current analysis, data show no evidence of an increased risk of developing uncommon or unexpected AEs, new or recurring malignancies, or diabetes during rhGH treatment. In both the US and international cohorts, less than 1% of reported SAEs were suspected to be treatment-related. The safety findings from this analysis are consistent with those from other post-approval registries of rhGH treatment [12, 15, 17–22].

rhGH doses were higher in patients from the USA compared with the international group. This has been observed previously, including in a comparative analysis of children treated with growth hormone in the real-world US ANSWER study and the European NordiNet® study [23]. In this analysis, growth hormone dosing at baseline and during treatment was higher in the US cohort compared with the European cohort, which was attributed to cultural differences in prescribing practices [23].

The use of higher rhGH doses in the US group compared with the international group may, in part, also be due to differences in the recommended rhGH starting doses. For example, the recommended starting dose for patients born SGA is up to 68 µg/kg/day in the USA [3] versus 35 µg/kg/day in Europe [2]. The recommended starting doses in other pediatric indications are similar in the USA and Europe [2, 3]. Less likely is that geographical differences exist even within approved dose ranges.

Adverse event data and growth response at Year 1, stratified by prescribed Omnitrope® dose at baseline, were assessed. Different cut-offs were chosen for the US (≤ 50 µg/kg/day, > 50 µg/kg/day) and international (≤ 35 µg/kg/day, > 35 µg/kg/day) groups, reflecting the different rhGH starting doses. In both cohorts, the proportion of AEs, SAEs, and treatment-related AEs was numerically higher in the high-dose than in the low-dose subgroup. Interestingly, for both cohorts, there was a little difference in the growth response between low and high baseline-dose groups.

Mean HSDS and HVSDS at baseline were higher in the US cohort compared with patients from the international group. This may reflect the fact that a higher proportion of US patients received rhGH treatment before entering PATRO Children (47%) compared with the international cohort (14%). When starting rhGH treatment in the PATRO Children study, US patients were on average older than patients in the international group, which may also be due to the higher proportion of pre-treated patients in the US group. Other possible explanations include delayed diagnosis and subsequent initiation of rhGH therapy in a larger number of US patients, and the higher proportion of ISS patients in the US cohort.

Further differences between the US and international cohorts include a higher proportion of males in the US group compared with the international group. Similar findings have been observed in other post-approval studies of rhGH [24]. A much larger percentage of US patients discontinued the study due to switching to another rhGH preparation; this is most likely explained by insurance-mandated changes. Insurance issues are known to be among the most common reasons for discontinuation of rhGH in the USA [25]. Other issues include insurance denial of coverage, or medication costs exceeding the capability to pay despite insurance coverage [25]. These factors are likely to contribute to the high proportion of US patients in PATRO Children who discontinued due to miscellaneous reasons.

As with all observational studies, there are several limitations to be considered. First, there is a risk of bias due to missing or erroneous information, as data are collected according to routine clinical practice. As patient visits are scheduled at the discretion of the treating physician and for patient convenience (rather than on a regular basis), there may be a long interval between visits for some, possibly causing AEs to be under-reported. The mean duration of observation on treatment in the study was relatively short (approximately 3 years) for both groups included in this analysis. This limits the interpretation of some data, for example, the occurrence of malignancies.

Another shortcoming is the difference in patient population between the two groups, with the US group comprising less than 5% of the international group. Furthermore, statistical comparisons between the groups were not pre-specified; hence, it was not possible to reliably assess the statistical significance of differences.

Based on this analysis of data from the PATRO Children study, Omnitrope® treatment is well tolerated and effective in US patients and those from other countries. Across the pediatric indications included, available data show no evidence of an increased risk of developing uncommon or unexpected AEs, new or recurring malignancies, or diabetes during rhGH treatment. Some differences were observed between the US and international groups, which likely reflect variations in referral patterns, rhGH treatment practices, and approved indications.

## Data Availability

The datasets generated during and/or analyzed during the current study are not publicly available, but are available from the corresponding author on reasonable request.

## Abbreviations

AE: Adverse event
BL: Baseline
BMI: Body mass index
CI: Confidence interval
CRI: Chronic renal insufficiency
eCRF: Electronic case report form
EMA: European Medicines Agency
GHD: Growth hormone deficiency
HSDS: Height standard deviation score
HV: Height velocity
HVSDS: Height velocity standard deviation score
ISS: Idiopathic short stature
MedDRA: Medical Dictionary for Regulatory Activities
NR: Not recorded
PATRO: PAtients TReated with Omnitrope®
PWS: Prader-Willi syndrome
rhGH: Recombinant human growth hormone
SAE: Serious adverse event
SD: Standard deviation
SDS: Standard deviation score
SGA: Small for gestational age
TS: Turner syndrome
UK: United Kingdom
US: United States
USA: United States of America
